# The Arbuscular Mycorrhizal Fungal Community Response to Warming and Grazing Differs between Soil and Roots on the Qinghai-Tibetan Plateau

**DOI:** 10.1371/journal.pone.0076447

**Published:** 2013-09-26

**Authors:** Wei Yang, Yong Zheng, Cheng Gao, Xinhua He, Qiong Ding, Yongchan Kim, Yichao Rui, Shiping Wang, Liang-Dong Guo

**Affiliations:** 1 State Key Laboratory of Mycology, Institute of Microbiology, Chinese Academy of Sciences, Beijing, China; 2 University of Chinese Academy of Sciences, Beijing, China; 3 School of Plant Biology, University of Western Australia, Crawley, Australia; 4 Laboratory of Alpine Ecology and Biodiversity, Institute of Tibetan Plateau Research, Chinese Academy of Sciences, Beijing, China; Dowling College, United States of America

## Abstract

Arbuscular mycorrhizal (AM) fungi form symbiotic associations with most plant species in terrestrial ecosystems, and are affected by environmental variations. To reveal the impact of disturbance on an AM fungal community under future global warming, we examined the abundance and community composition of AM fungi in both soil and mixed roots in an alpine meadow on the Qinghai-Tibetan Plateau, China. Warming and grazing had no significant effect on AM root colonization, spore density and extraradical hyphal density. A total of 65 operational taxonomic units (OTUs) of AM fungi were identified from soil and roots using molecular techniques. AM fungal OTU richness was higher in soil (54 OTUs) than in roots (34 OTUs), and some AM fungi that differed between soil and roots, showed significantly biased occurrence to warming or grazing. Warming and grazing did not significantly affect AM fungal OTU richness in soil, but warming with grazing significantly increased AM fungal OTU richness in roots compared to the grazing-only treatment. Non-metric multidimensional scaling analysis showed that the AM fungal community composition was significantly different between soil and roots, and was significantly affected by grazing in roots, whereas in soil it was significantly affected by warming and plant species richness. The results suggest that the AM fungal community responds differently to warming and grazing in soil compared with roots. This study provides insights into the role of AM fungi under global environmental change scenarios in alpine meadows of the Qinghai-Tibetan Plateau.

## Introduction

Arbuscular mycorrhizae (AM) are symbiotic associations between plant roots and soil fungi of the Phylum Glomeromycota [1]. In the AM association, the plant provides photosynthetic carbon for the growth and function of AM fungi, and the plant performance could thus affect AM fungal community [1–3]. In turn, AM fungi supply soil nutrients to host plants and hence can exert strong effects on plant communities [1,4,5] and consequently affect ecosystem processes [6]. It is accepted that AM associations, as critical links between the above- and belowground biotic communities in ecosystems, are affected by environmental variations [3,7,8]. In particular, with the unprecedented magnitude of global temperature increase associated with anthropogenic activities, it is of great concern how the AM fungal community responds to disturbance under climate change scenarios in natural ecosystems [3,9].

Temperature manipulation studies have shown that warming affects not only plant productivity, diversity and community composition [10,11], but also AM fungal community structure and function in ecosystems [12,13]. For example, it has been demonstrated that effects of warming on AM root colonization can be positive [13–15], negative [16] or neutral [12,17,18]. In addition, warming showed positive effects on extraradical hyphal (ERH) density [14,17] and spore density [15], but a negative effect on vesicle density [18] or no effect on AM fungal community composition [12]. Such varying observations suggest that AM fungi do not always respond consistently to temperature variation.

As one of the major land uses of natural grasslands, livestock grazing has affected plant primary production, species composition, soil nutrient cycling [10,11] and AM fungi [19,20]. However, the effects of grazing on AM fungal community yield inconsistent conclusions. For instance, grazing had positive [19,21,22] and no or negative [23,24] effects on AM root colonization. Meanwhile, grazing increased AM fungal abundance and species richness in a temperate grassland of Yellowstone National Park, USA [25], but decreased AM fungal spore density in temperate grasslands in Argentina [26] and in Inner Mongolia of China [27]. Although effects of the warming-only or grazing-only treatment on AM fungal community have received considerable attention in soil [19,20,25,27,28] or roots [12], to our knowledge, the combined effect of warming and grazing on AM fungal communities in both soil and roots has not been documented in natural ecosystems.

Accurate AM identification is important in order to understand AM fungal diversity in natural ecosystems [29]. Since AM fungal spore occurrence and morphology cannot reveal a symbiotically active organism community [30], molecular techniques have thus been developed to overcome the limitation of morphological identification [31–33]. For example, primers that target the internal transcribed spacer (ITS) region, large subunit (LSU) gene and small subunit (SSU) gene of rDNA have been frequently used to detect AM fungal communities in ecosystems [32–35]. However, the ITS region, LSU or SSU gene alone is unable to resolve closely related AM fungal species [31]. Recently, new primer pairs SSUm-Af/LSUm-Ar combined with SSUm-Cf/LSUm-Br have been developed to amplify ~1,500 bp fragment spanning SSU, ITS and LSU of Glomeromycota members, the best discrimination within AM fungal species as well as against non-AM fungi and plants [29,36,37].

The Qinghai-Tibetan Plateau covers 2.5 million km^2^ in China, and is dominated by alpine meadow, which is sensitive to climate change and anthropogenic activities [10]. A controlled warming-grazing experimental system has therefore been established in the alpine meadow ecosystem to study the responses of plants, bacteria, soil properties and carbon dynamics to warming and grazing [11,38–40]. However, knowledge of how the AM fungal communities respond to warming and grazing is limited in this alpine meadow ecosystem on the Qinghai-Tibetan Plateau.

To better understand the effects of warming and grazing on AM fungal communities, we studied AM root colonization, ERH density and spore density in root and soil samples from a 3-year warming-grazing alpine meadow on the Qinghai-Tibetan Plateau. The AM fungal community composition was also examined in both mixed roots and soil using the SSU-ITS-LSU fragment as an AM fungal barcode [29,36,37]. In this alpine meadow ecosystem, we tested the following three hypotheses: H_1_, the AM root colonization, ERH density and spore density in mixed roots and soil will be affected by warming and grazing; H_2_, the AM fungal community differs between soil and mixed roots; and H_3_, the response of the AM fungal community to warming and/or grazing will differ between soil and roots. The outcome could provide insights into our understanding of the role of AM fungi under global environmental change scenarios in alpine ecosystems.

## Materials and Methods

### Ethics statement

The Haibei Alpine Meadow Ecosystem Research Station (HBAMERS) is run by the Northwest Institute of Plateau Biology, Chinese Academy of Sciences. No specific permits are required for the described field studies. The study sites are not privately-owned or protected in any way, and the field studies did not involve endangered or protected species.

### Study site

The study was conducted at the HBAMERS, northeast Qinghai-Tibetan Plateau, China (37°37’ N and 101°12’ E, 3,200 m above sea level). This site has a typical plateau continental climate with a short and cool summer and a long and severely cold winter. Annual mean temperature is -2°C, and annual mean precipitation is 500 mm (

> 80% during the summer monsoon season). The plant community at the experimental site is dominated by *Kobresia humilis*, *Festuca ovina*, *Elymus nutans*, *Poa pratensis*, *Carex scabrirostris*, *Scirpus distigmaticus*, *Gentiana straminea*, *Gentiana farreri*, *Blysmus sinocompressus*, and *Potentilla nivea*. The soil is classified as a Mat-Gryic Cambisol [11].

### Controlled warming-grazing experiment and sampling

A controlled warming-grazing experiment was established in the HBAMERS in 2006 [38]. Briefly, the infrared heating system (a free-air temperature enhancement, FATE) was employed according to Kimball et al. [41]. The heaters were controlled by a proportional-integral-derivative-outputs (PID) system to ensure constant warming between the heated and un-heated reference plots. The set point differences of the vegetation between heated and reference plots were 1.2°C during daytime and 1.7°C at night. In a two-way factorial design (warming and grazing) there were four treatments: (1) no-warming with no-grazing (as a control, C), (2) warming with no-grazing (W) (3), no-warming with grazing (G), and (4) warming with grazing (WG). Each treatment had four replicates. A total of 16 plots (3 m diameter each and 3 m separation from each other) were in a randomized arrangement.

A moderate grazing intensity was set as follows. One adult Tibetan sheep was initially fenced in the morning of 15 August 2006 for ~2 h in each grazing plot, where the vegetation height was ~8–9 cm and ~4–5cm before and after grazing, respectively. Two adult Tibetan sheep were later fenced in the morning of 12 July, 3 August and 12 September 2007, 8 July and 20 August 2008, and 9 July and 24 August 2009 for ~1 h in each grazing plot, where the vegetation height was ~6–7 cm and ~3–4 cm before and after grazing, respectively.

On 2 August 2009 (23 days after grazing), five soil cores (30 cm depth, 1.8 cm diameter) from each plot were randomly collected and mixed as one composite sample. Fresh soil samples were sieved (1-mm sieve) to remove roots and debris. Fine roots (

< 1 mm diameter) were manually collected, washed with sterilized deionized water, and blotted dry on filter paper. All fresh root and part soil samples were then stored at −80°C until further AM fungal analyses. The remaining soil samples were used to measure soil variables including pH, soil moisture (SM), soil organic carbon (SOC), soil organic nitrogen (SON), total phosphorus (TP) and total nitrogen (TN) by Rui et al. [39]. Plant variables including aboveground net primary production (ANPP) and plant species richness at the end of August have been determined by Wang et al. [11]. Information on these soil and plant variables is presented in Table S1.

### AM root colonization, ERH density and spore density

Fifty fine root fragments (*ca* 1 cm long) of each sample were stained with acid fuchsin and the percentage of AM root colonization was quantified by the magnified line-intersect method [42]. Extraction of fungal hyphae followed Rillig et al. [43] with modifications. Briefly, 4.0 g fresh soil was suspended with 100 ml deionized water and 12 ml sodium hexametaphosphate (35 g l^-1^). The soil suspension was then blended for 30 s and settled for 30 min. The supernatant was poured through a 38-µm sieve to retain hyphae, roots and other particles, and the hyphae were gently transferred into a flask with deionized water until a final volume of 200 ml. The flask was then shaken manually for 5 s, and 2 ml was then pipetted onto a 25-µm filter (Xingya, China). The filter was then dyed with 1% acid fuchsin and observed under 200 × magnification (Nikon 80i, Japan). Hyphae were distinguished into mycorrhizal and non-mycorrhizal hyphae based on their morphology and staining color according to Miller et al. [44]. AM fungal spores were extracted from 20.0 g air-dried soil of each sample with deionized water using the wet-sieving and decanting method and counted under 50 × magnification [45].

### Molecular analysis of AM fungi

Genomic DNA was extracted from 100.0 mg frozen roots with a DNeasy Plant Mini Kit (Qiagen, Crawley, UK) or from 5.0 g frozen soil with a PowerMax Soil DNA Isolation Kit (MoBio Laboratories, Inc., Carlsbad, CA, USA) following the manufacturers’ instructions. The primer pairs SSUm-Af/LSUm-Ar and SSUm-Cf/LSUm-Br were used for the first and nested PCR, respectively [29]. The nested purified PCR products were transformed into *E. coli* JM109 for white and blue screening. For each library, ~90 positive colonies were picked and grown overnight in liquid Luria-Bertani (LB) medium. Then PCR was carried out using 1 µl liquid culture of *E. coli* as templates with the primer pair T7 and SP6, and the restriction fragment length polymorphism (RFLP) was performed in a 10 µl reaction system with the *Mbo*I and *Hinf*I (Fermentas, USA). One representative of each PCR-RFLP type from each clone library was then sequenced with an ABI Prism 3700 Genetic Analyzer (Applied Biosystems, USA).

The obtained sequences were proofread and trimmed to remove the vector sequence with the SEQMAN program in the LASERGENE software Package (DNA Star Inc., Madison, WI, USA), and then compared with sequences in the GenBank [46]. Sequences displaying 92–93% similarity were usually treated as the same OTU for ITS [47], and 97% for the partial SSU and LSU gene [3,32,33] in previous studies. Therefore, the SSU-ITS-LSU fragment (~1,500 bp) sequences were grouped into the same OTU with a 93% sequence similarity using the Sequencher 4.80 (Gene Codes Corporation, Ann Arbor, Michigan, USA). We picked one RFLP type as a representative sequence for the OTU. Then the sequences of obtained OTUs and the reference sequences of Glomeromycota from the GenBank were aligned using the Mafft-7.017 [48]. The Bayesian (GTR+I+G model) and neighbor-joining (the Kimura 2-parameter model with 1000 bootstrap replications) phylogenetic analyses were performed using the MrBayes 3.1 [49] and PAUP4.0 [50], respectively. Bayesian posterior probabilities (BPP) were obtained from the 50% majority rule consensus trees generated by 1,000,000 generations with 250,000 “burnin”. The trees were rooted with *Paraglomuslaccatum* . The AM fungal OTUs were assigned to different families based on the phylogenetic tree. Members of Glomerales were separated into the Glomus Group A (Glomeraceae) and Glomus Group B (Claroideoglomeraceae), and *Glomus* group C (Diversisporaceae) [51,52]. All sequences obtained in this study have been deposited in the GenBank with accession numbers JX096566-JX096630.

### Data analysis

Abundance of a given AM fungal OTU is defined as clone numbers of that OTU in a sample, and abundance of a given family is the sum of abundance of all OTUs belonging to that family in a sample. Richness of a given family is all OTU numbers of that family in a sample. Frequency of a given AM fungal OTU is defined as the occurrence of that species in all samples. The AM fungal OTUs which occurred in more than three samples (frequency > 18.7%) from either soil or roots were defined as the common OTUs.

A two-way ANOVA was used to test the effects of warming, grazing and their interaction on AM root colonization, ERH density, spore density, OTU richness, OTU abundance, and family abundance. All data were tested for normality and homogeneity of variance before two-way ANOVA. Of these data, only the abundance of OTU25 in roots did not meet the normal distribution before and after transformation, and then the Tamhane’s T2 post hoc was applied using the original data. The other variables were then compared among treatments using Tukey’s HSD tests in SPSS 17.0 software. The difference in the abundance of AM fungi between soil and roots was assessed by the paired t-test. In order to assess the efficiency of the clone library, rarefaction curves were constructed for each treatment using the Estimate S 8.0 [53].

Both in soil and roots, distance matrices of AM fungal community (sequence number dataset, wisconsin-sqrt transformed) were calculated by the Bray-Curtis dissimilarity, and then subjected to non-metric multidimensional scaling (NMDS) ordinations. Using the ‘*envfit*’ function of the Vegan package with 999 permutations [54] in R [55], the treatments were fitted as centroids onto the ordination graphs, and the soil (SOC, SON, TN, TP, SM and pH) and plant (species richness and ANPP) variables were fitted as vectors onto the ordination graphics to understand if the AM fungal community composition was affected by one of these variables.

## Results

### AM root colonization, ERH density and spore density

There were no effects of warming (W), grazing (G) and their interaction (W×G) on AM root colonization (W:F = 0.29, *P* = 0.60; G: *F* = 0.00, *P* = 0.96; W×G: *F* = 5.39, *P* = 0.052), spore density (W:F = 3.12, *P* = 0.10; G: *F* = 0.10, *P* = 0.34; W×G: *F* = 3.21, *P* = 0.10) and ERH density (W:F = 0.02, *P* = 0.88; G: *F* = 0.02, *P* = 0.89; W×G: *F* = 0.09, *P* = 0.77). AM root colonization ranged from 34.8 ± 7.2% to 45.1 ± 5.7%, spore density from 13.6 ± 4.2 to 30.8 ± 15.3 (spore g^-1^ DW), and ERH density from 1.27 ± 0.16 to 1.34 ± 0.37 (m g^-1^ DW) amongst the C, W, G and WG treatments (means ± SD, n = 4).

### Comparison of AM fungal community between soil and roots

A total of 2,560 positive clones were obtained from the 32 clone libraries (16 from soil and 16 from roots). Subsequently, 640 RFLP types (380 from soil and 260 from roots) from these 2,560 clones screened by the *Hinf*I and *Mbo*I were sequenced. The BLAST search in GenBank indicated that 632 clones (98.8% of the total clones) were of AM fungal origin, and the other eight clones belonged to non-AM fungi (4 clones in Chytridiomycota, 2 in Basidiomycota, and 2 in Zygomycota). These 632 AM fungal sequences were grouped into 65 OTUs according to the 93% similarity threshold and identified as different taxonomic groups based on the phylogenetic analyses (Figure S1, Table S2). Among the 65 OTUs, 54 were from soil, 34 from roots, and 23 from both soil and roots (Figure S1). Of the 54 OTUs from soil, 42 belonged to Glomeraceae (Glomus Group A), 3 to Claroideoglomeraceae (Glomus Group B), 5 to Diversisporaceae, 3 to Gigasporaceae and 1 to Ambisporaceae. Of the 34 OTUs from roots, 25 belonged to Glomeraceae (Glomus Group A), 1 to Claroideoglomeraceae (Glomus Group B), 1 to Diversisporaceae and 7 to Gigasporaceae (Table S2). A rarefaction analysis indicated that the sampling effort was sufficient to identify the major AM fungi from soil and roots (Figure S2).

**Figure 1:**
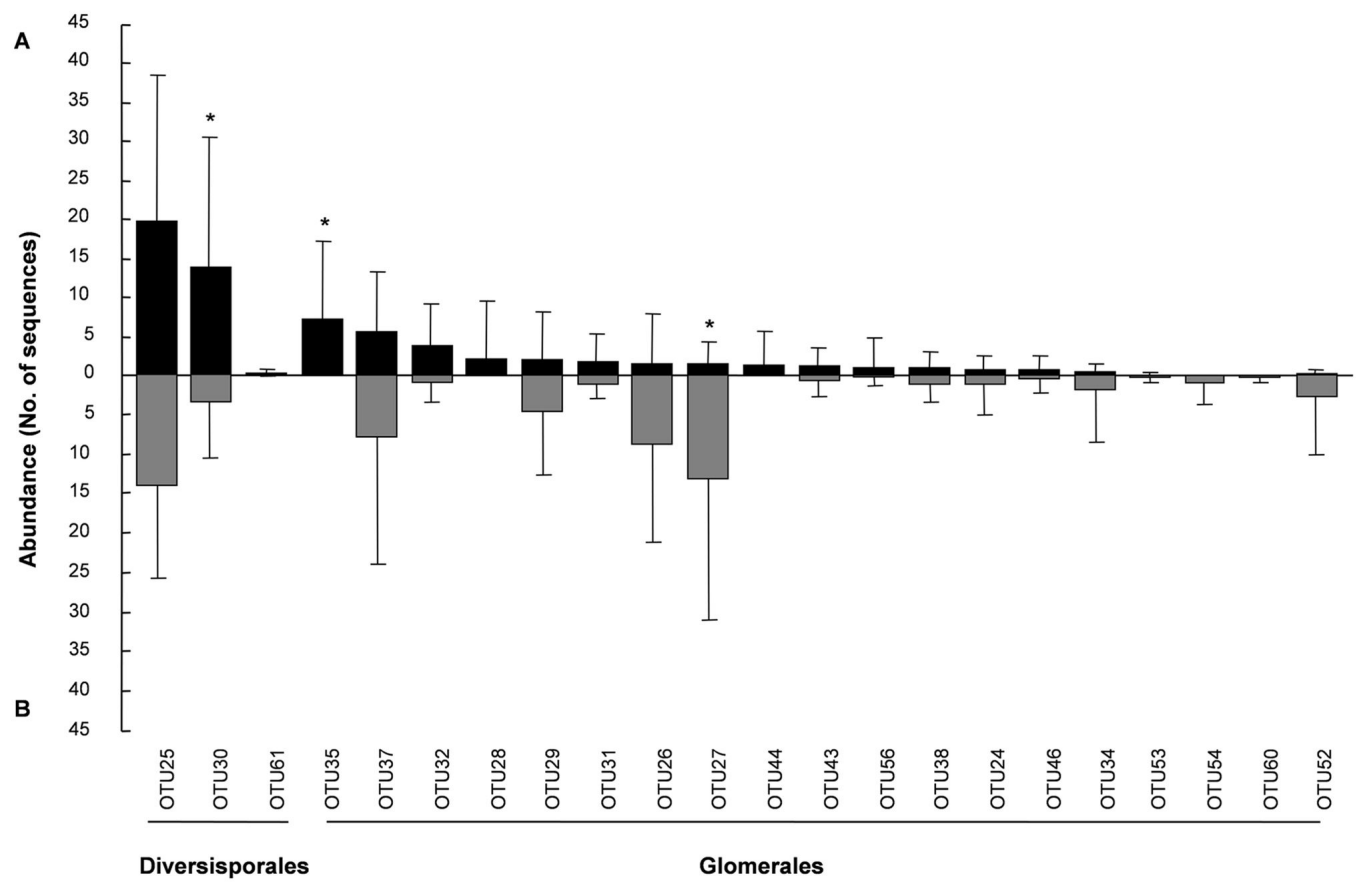
Common AM fungal OTUs (frequency > 18.7%) recovered from (A) soil and (B) roots. * indicates significant differences in the abundance of AM fungal OTUs between soil and roots according to the paired t-test at *P* < 0.05. Bars are standard deviation of the means (n = 16).

**Figure 2:**
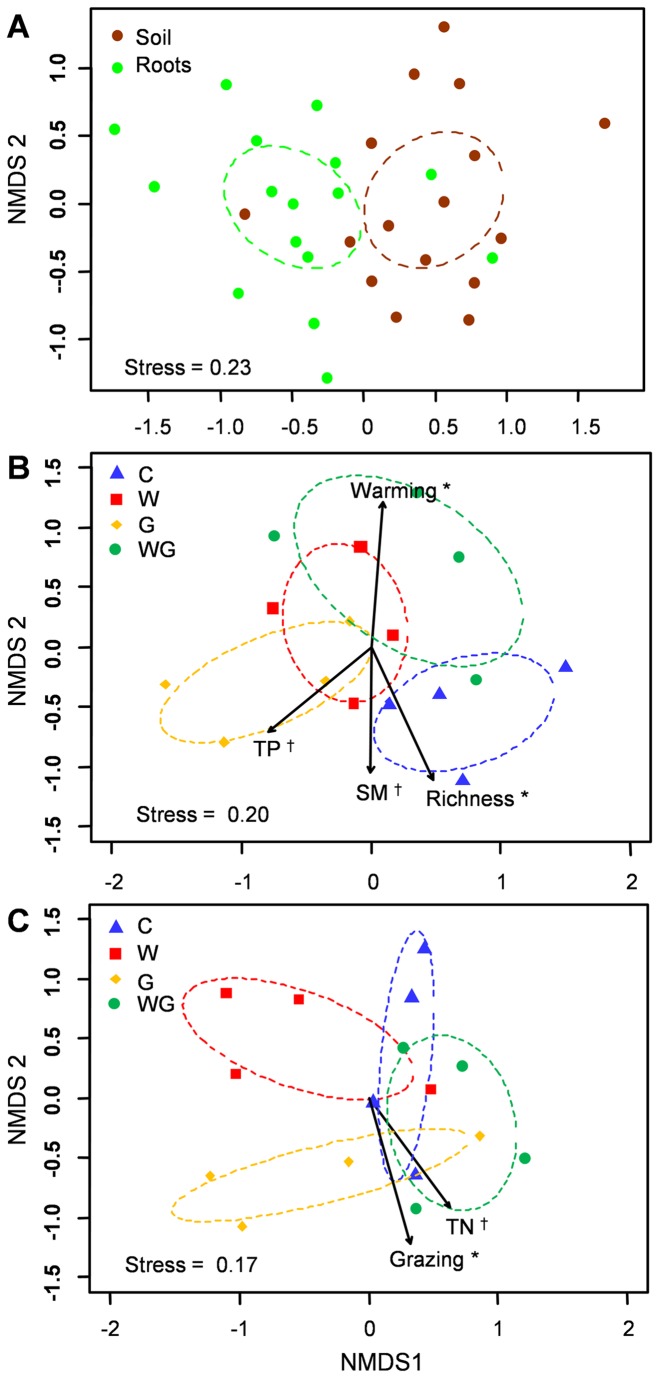
Non-metric multidimensional scaling (NMDS) of AM fungal community composition. AM fungal community composition (A) between soil and roots, (B) in soil among the four treatments, and (C) in roots among the four treatments. The treatments were fitted as centroids onto ordination graphics. Soil and plant variables were fitted as vectors onto ordination graphics. ^†^0.05 < *P* < 0.10, *P < 0.05. Abbreviations or terms: Richness, plant species richness; SM, soil moisture; TN, total nitrogen; TP, total phosphorus; C, no-warming with no-grazing; W, warming with no-grazing; G, no-warming with grazing; WG, warming with grazing.

A total of 22 common AM fungal OTUs (frequency > 18.7%) were determined in soil and roots (Figure 1). Among the 22 common OTUs, the abundance of OTU30 (Diversisporaceae) and OTU35 (Glomeraceae) were lower in roots than in soil (*P* < 0.05). However, the abundance of OTU27 (Glomeraceae) was higher in roots than in soil (*P* < 0.05). The NMDS analysis indicated that the AM fungal community composition was different between soil and roots (*r* ^2^ = 0.39, *P* = 0.002, Figure 2A).

### Responses of AM fungal communities in soil and roots to warming and grazing

Of the 54 AM fungal OTUs present in the soil, 18 (23.8% of total clone sequences) were found in C, 30 (25.8%) in W, 24 (24.5%) in G, 28 (25.9%) in WG, and five OTUs were recorded in all four treatments (Table S2). Of the 34 AM fungal OTUs present in roots, 20 (26.6% of total clone sequences) were found in C, 14 (24.9%) in W, 9 (22.1%) in G, 18 (26.6%) in WG, and 4 OTUs were shared among the four treatments (Table S2).

**Figure 3:**
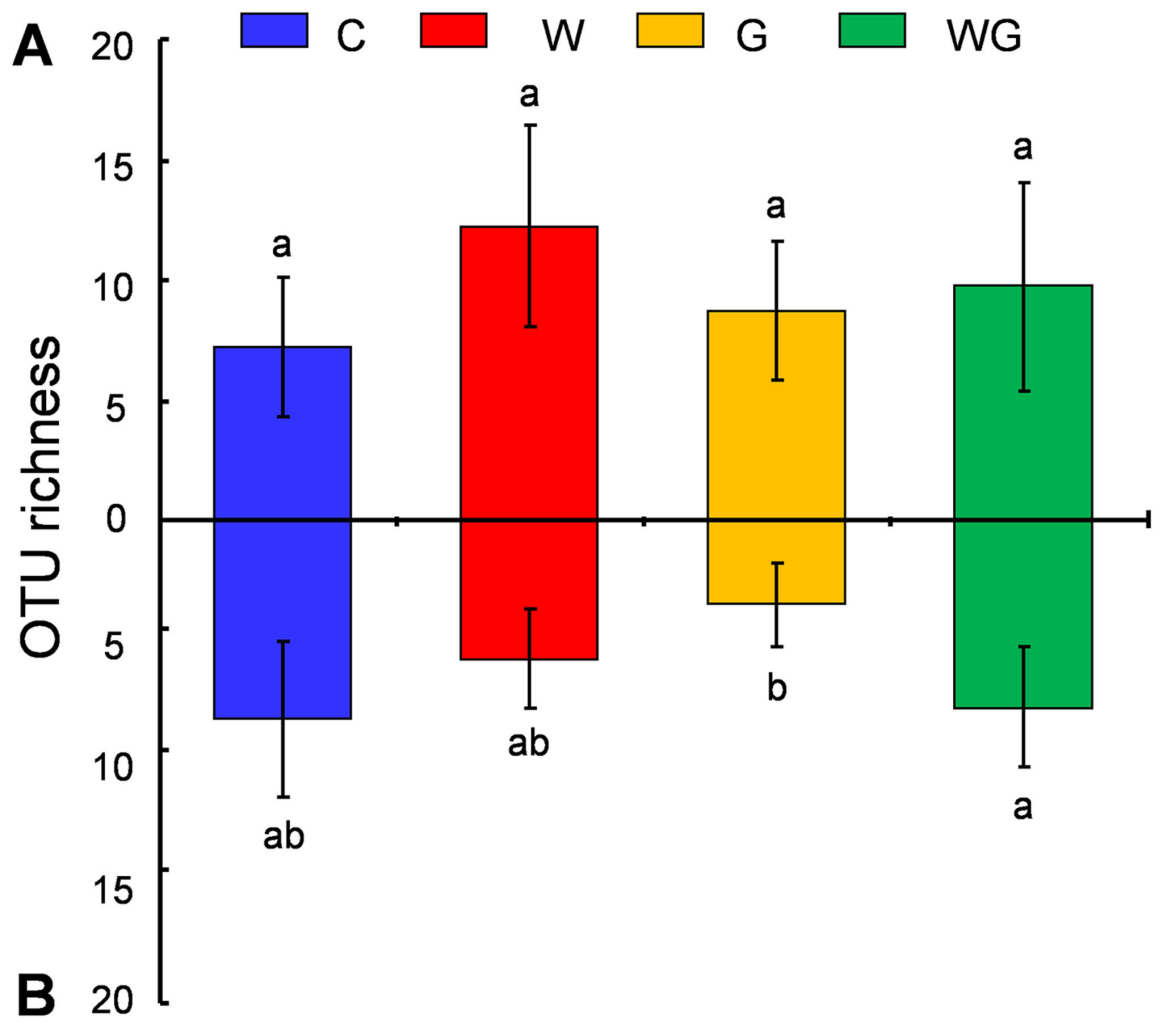
AM fungal OTU richness (A) in soil and (B) in roots among the four treatments. Bars without shared letters indicate significant difference at *P* < 0.05. Bars are standard deviations of the means (n = 4). Abbreviations: C, no-warming with no-grazing; W, warming with no-grazing; G, no-warming with grazing; WG, warming with grazing.

**Figure 4:**
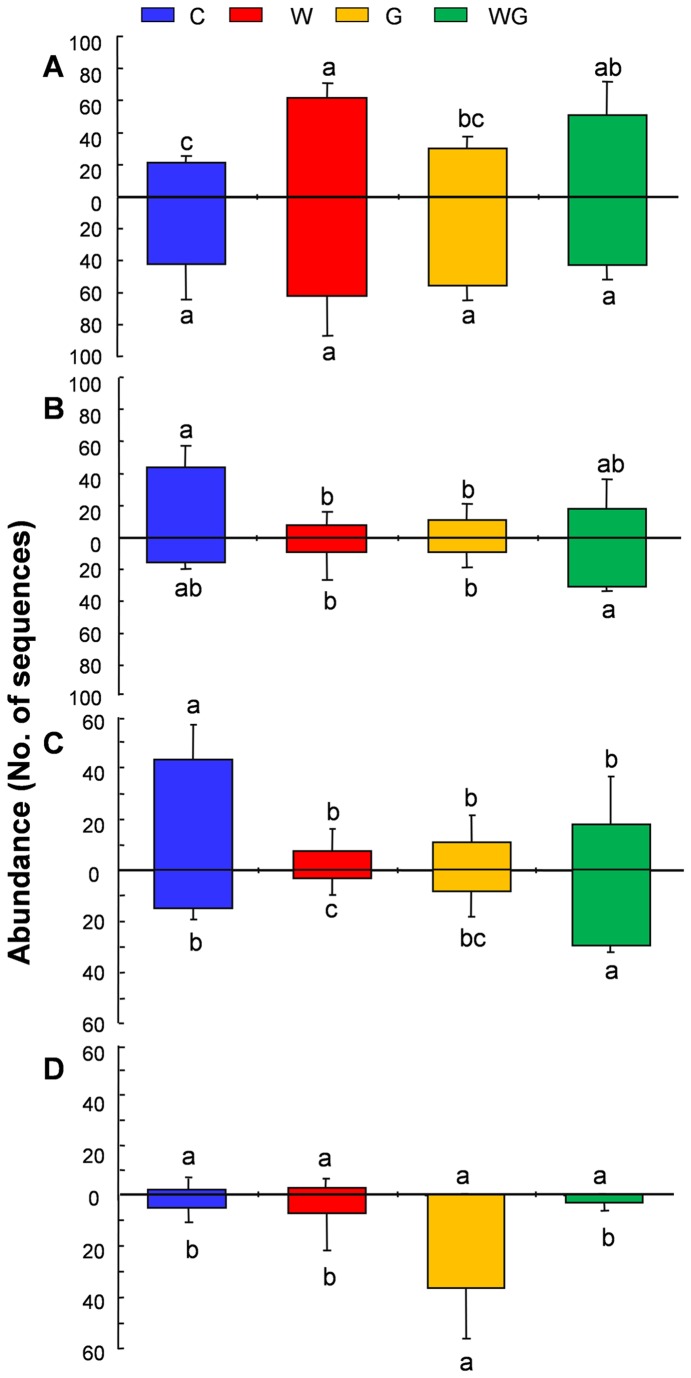
Abundance of common families and OTUs in soil and roots among the four treatments. A: Glomeraceae in soil (above X-axis) and roots (below X-axis); B: Gigasporaceae in soil (above X-axis) and roots (below X-axis); C: OTU25 in soil (above X-axis) and roots (below X-axis); D: OTU27 in soil (above X-axis) and roots (below X-axis). Bars without shared letters indicate significant differences at *P* < 0.05. Bars are standard deviations of the means (n = 4). Abbreviations: C, no-warming with no-grazing; W, warming with no-grazing; G, no-warming with grazing; WG, warming with grazing.

Neither warming nor grazing had a significant effect on AM fungal OTU richness in soil (*P* > 0.05, Figure 3A). However, there was a significant additive effect of the WG treatment compared to the grazing-only treatment on AM fungal OTU richness in roots (*F* = 7.67, *P* < 0.05), i.e. WG significantly increased AM fungal OTU richness by 122.9% compared with the grazing alone in roots (Figure 3B). Meanwhile, warming, grazing and their interaction had no significant effects on the AM fungal OTU richness of each family in both soil and roots (*P* > 0.05, Table S3). On the other hand, a warming effect (*F* = 25.7, *P* < 0.01) was observed on the abundance of Glomeraceae in soil, and warming alone significantly increased its abundance by 187.2% compared with the control treatment (Figure 4A). By contrast, only an interactive effect between warming and grazing was observed on the abundance of Gigasporaceae in both soil (*F* = 10.5, *P* < 0.01) and roots (*F* = 7.7, *P* < 0.05). For example, the abundance of Gigasporaceae in soil was significantly decreased by warming alone (81.8%) and grazing alone (75%) compared with the control treatment; whereas in roots WG significantly increased Gigasporaceae in abundance by 248.6% and 248.6% compared with warming alone and grazing alone, respectively (Figure 4B).

Among the 22 common AM fungal OTUs (frequency > 18.7%), ANOVA results indicated that OTU25 (Gigasporaceae) and OTU27 (Glomeraceae) showed a biased occurrence among the four treatments (*P* < 0.01, Figure 4) in soil or roots. For example, the abundance of OTU25 in soil was significantly decreased by warming alone (82.6%), grazing alone (74.4%) and WG (58.7%) compared with the control treatment (Figure 4C). In contrast, the abundance of OTU25 in roots was significantly decreased by warming alone (78.3%) compared with the control, but significantly increased by WG compared with the control (96.7%), grazing alone (247.1%) and warming alone (807.7%) treatments (Figure 4C). Although OTU27 in soil did not show significantly biased occurrence among treatments, the grazing-only treatment significantly increased the abundance of OTU27 in roots by 590.5% compared with all other treatments (Figure 4D).

NMDS analyses indicated that the AM fungal community compositions were affected by the treatments in both soil (*r* ^2^ = 0.53, *P* < 0.01, Figure 2B) and roots (*r* ^2^ = 0.44, *P* < 0.01, Figure 2C). Furthermore, the AM fungal community composition in soil was related to warming (*r* ^2^ = 0.44, *P* < 0.05) and plant species richness (*r* ^2^ = 0.43, *P* < 0.05), and marginally related to soil moisture (*r* ^2^ = 0.32, *P* = 0.09) and total P (*r* ^2^ = 0.33, *P* = 0.08, Figure 2B). However, the AM fungal community composition in roots was related to grazing (*r* ^2^ = 0.43, *P* < 0.05) and marginally related to total N (*r* ^2^ = 0.33, *P* = 0.07, Figure 2C).

## Discussion

A 3-year warming and grazing treatment did not significantly affect AM root colonization, spore density or ERH density in an alpine meadow on the Qinghai-Tibetan Plateau, which did not support our first hypothesis (H_1_). Similarly, AM root colonization was not significantly affected by a 1-year-period warming in grassland ecosystems in York, UK [12] and in California, USA [17] or by a 2- or 4-year warming in *Bouteloua* *gracilis* in Colorado, USA [16]. Grazing also had no significant effect on AM fungal spore density in a mountain grassland ecosystem in Argentina, South America [26]. A meta-analysis of 33 publications demonstrated that grazing had generally only decreased AM root colonization by 3% [24]. However, AM root colonization and ERH density were significantly increased by a range of warming (5 to 14°C) in greenhouse studies [14,56]. In addition, AM fungal spore density was significantly affected by a 20-year grazing experiment in Inner Mongolia steppe, China [27] and a 40-year grazing experiment in Yellowstone National Park, USA [25]. These inconsistent results suggest that AM fungal abundance may have different responses to intensity or duration of warming and grazing, with an overall increased response among the longer running experiments.

The AM fungal community was significantly different between soil and roots in this alpine meadow ecosystem as was expected (H_2_, Figure 2A). The conspicuous difference in AM fungal community composition between soil and roots has been reported previously [3,34,57–61]. In addition, some AM fungi had a noticeably biased occurrence in soil or roots, for example, OTU30 (Diversisporaceae) and OTU35 (Glomeraceae) favoured soil, whereas OTU27 (Glomeraceae) was abundant in roots. It has been suggested that the phenology of AM fungi may generate distinct root and soil communities, which may help partition fungal niches in time and space [3,62]. Alternatively, there may be different ecological and evolutionary forces for structuring soil AM fungal community compared with roots [3]. Furthermore, we found much higher AM fungal richness in soil (54 OTUs) than in roots (34 OTUs). The consistent result of higher AM fungal diversity in soil than in roots has been also demonstrated in previous studies [3,34,58–60]. This may be explained by the seasonal nature of AM fungal communities [60,63,64]. In addition to AM fungal propagules of current symbionts, formerly active symbionts could remain in soil compared to the roots [60,64].

The AM fungal community between soil and roots responded differently to warming and grazing as was also expected (H_3_, Fig. 2B, 2C). The AM fungal community composition in soil was significantly related to warming and plant species richness (Fig. 2B, Table S1). There was an decrease in plant species richness with warming [11], which are thus co-relating factors affecting the AM fungal community; thus lower plant richness with warming may result in a change of AM fungal community composition [2,3]. In addition, the AM fungal community composition in soil was marginally related to soil moisture and total P (Fig. 2B), which are crucial factors in determining the AM fungal community composition [1,65,66]. However, the AM fungal community composition in roots was significantly related to grazing, but not to warming in this alpine meadow (Fig. 2C). The results of the present study were consistent with previous studies that AM fungal composition was significantly affected by grazing in a temperate grassland in the USA [20], but not by warming in a native grassland in UK [12]. It is possible that grazing changes the allocation of carbohydrates to roots [67,68], which may result in a change in the AM fungal community composition. In addition, the AM fungal community composition in roots was marginally related to total N (Figure 2C), in agreement with previous studies, showing that nitrogen may affect the patterns of AM fungal communities [3,69].

Warming and grazing had no significant effect on the AM fungal OTU richness of each family in soil and roots (Table S3). Although warming effect on AM fungal families was not reported so far, studies of other global change effects on AM fungal families showed the similar trends for Glomeraceae. For instance, Klironomos et al. [70] found that richness of Glomeraceae was not affected by elevated CO_2_ levels, but the richness of Gigasporaceae declined in soil. In addition, the abundance of Glomeraceae, Gigasporaceae and some OTUs showed different responses to warming and grazing in soil and roots (Figure 4). Several studies also found that AM fungal species varied their responses to elevated CO_2_ [71], grazing [26] and N enrichment [69]. As there are different colonization strategies between Glomeraceae and Gigasporaceae [72], it is likely that AM fungal OTUs that belong to different families possess functionally diverse traits [73]. However, most AM fungal OTUs did not show significantly biased occurrence to warming and grazing in this alpine meadow ecosystem. For example, the abundance of OTU26, which is closely related to the globally widespread *Rhizophagusintraradices* (= *Glomus intraradices*) (Figure S1) [52,64,69,74], was not significantly affected by warming and grazing in soil and roots. Similarly, the abundance of *R* *. intraradices* was not affected by elevated CO_2_ in a successional grassland [71] but marginally decreased by simulated N deposition in hardwood forests of Michigan, USA [69]. Thus, it is possible that *R* *. intraradices* shows a wide tolerance to environmental stress or there is functional diversity within this species [64,74].

Although the effect of the warming-only [12] or grazing-only [19,25,27,28] treatment on AM fungal community has been documented in ecosystems, this study is the first to investigate the combined effect of warming and grazing on the AM fungal community structure in an alpine meadow ecosystem on the Qinghai-Tibetan Plateau. Our results showed no significant interactive effects between warming and grazing on AM root colonization, ERH density and spore density. In contrast, previous studies showed significant interactive effects between warming and elevated CO_2_ on AM fungal ERH density [56] and between warming and moisture on AM root colonization [75]. Furthermore, our results did demonstrate significant interactive effects between warming and grazing on the abundance of some AM fungal OTUs and families (Figure 4). Significant interactive effects observed in this and previous studies suggest that AM fungi may demonstrate complex responses under multiple global change factors in ecosystems [56].

In conclusion, the AM fungal community composition was different between soil and roots, and AM fungal OTU richness was higher in soil than in roots in this alpine meadow ecosystem as reported in other studies [34,57–59]. The AM fungal community thus responds differently to warming and grazing in soil versus roots. These results not only provide new information about how AM fungi respond to abiotic environmental stress, but also enhance our understanding of the role of AM fungi under global climate change scenarios in an alpine meadow ecosystem on the Qinghai-Tibetan Plateau. Nevertheless, future studies are warranted to identify seasonal and/or yearly responses of AM fungal communities to long-term exposure of warming and grazing since the results of this study have been only derived from an annual (summer) sampling in a 3-year-period of warming and grazing in this alpine meadow ecosystem.

## Supporting Information

Figure S1 **Phylogenetic tree based on ~1,500 bp fragment of Glomeromycota from soil (circles) and roots (triangles).** The tree is rooted with *Paraglomuslaccatum* . The GenBank accession numbers are placed in parentheses after OTUs. The numbers at each branch point (e.g. 100/100) represent bootstrap support calculated from 1,000 replicates (left) and Bayesian posterior probabilities (right). * indicates lack of support for a particular clade or value < 50%. Bar indicates 0.1 expected changes per site.
(PDF)

Figure S2 **Rarefaction curves for AM fungal OTUs obtained from soil and roots in A: no-warming with no-grazing; B: warming with no-grazing; C: no-warming with grazing; D: warming with grazing.**
(TIF)

Table S1 **Aboveground net primary production (ANPP, g m^-2^), plant species richness (Richness), soil moisture (SM, %), soil organic carbon (SOC, mg kg^-1^ DW), soil organic nitrogen (SON, mg kg^-1^ DW), total nitrogen (TN, % DW), total phosphorus (TP, mg kg^-1^ DW), and pH value after a 3-year-period of warming and/or grazing.**
(DOCX)

Table S2 **Abundance (sequence numbers) of arbuscular mycorrhizal (AM) fungal OTUs in soil and roots under no-warming with no-grazing (C), warming with no-grazing (W), no-warming with grazing (G), and warming with grazing (WG).**
(DOCX)

Table S3 **Arbuscular mycorrhizal fungal richness of Glomeraceae, Claroideoglomeraceae, Diversisporaceae and Gigasporaceae in soil and roots under no-warming with no-grazing (C), warming with no-grazing (W), no-warming with grazing (G), and warming with grazing (WG).**
(DOCX)
