# Supplementary figures and images for: The Arbuscular Mycorrhizal Fungal Community Response to Warming and Grazing Differs between Soil and Roots on the Qinghai-Tibetan Plateau
Source: PLoS One. 2013 Sep 26;8(9):e76447. doi: 10.1371/journal.pone.0076447 (PMC3784447; doi:10.1371/journal.pone.0076447)

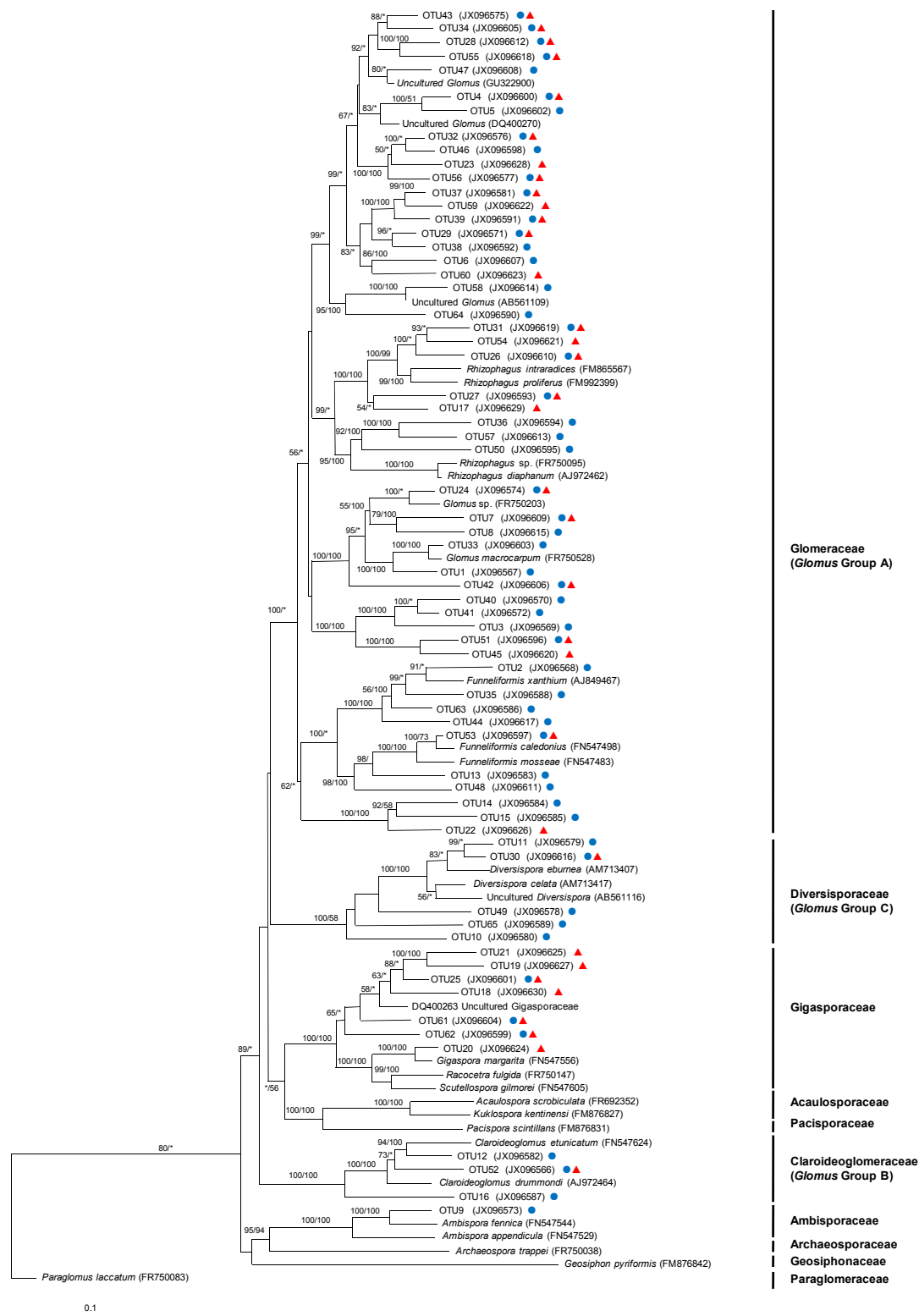

Supplement: Figure S1 — Phylogenetic tree based on ~1,500 bp fragment of Glomeromycota from soil (circles) and roots (triangles). The tree is rooted with Paraglomuslaccatum . The GenBank accession numbers are placed in parentheses after OTUs. The numbers at each branch point (e.g. 100/100) represent bootstrap support calculated from 1,000 replicates (left) and Bayesian posterior probabilities (right). * indicates lack of support for a particular clade or value < 50%. Bar indicates 0.1 expected changes per site. (PDF) [file pone.0076447.s001.pdf]

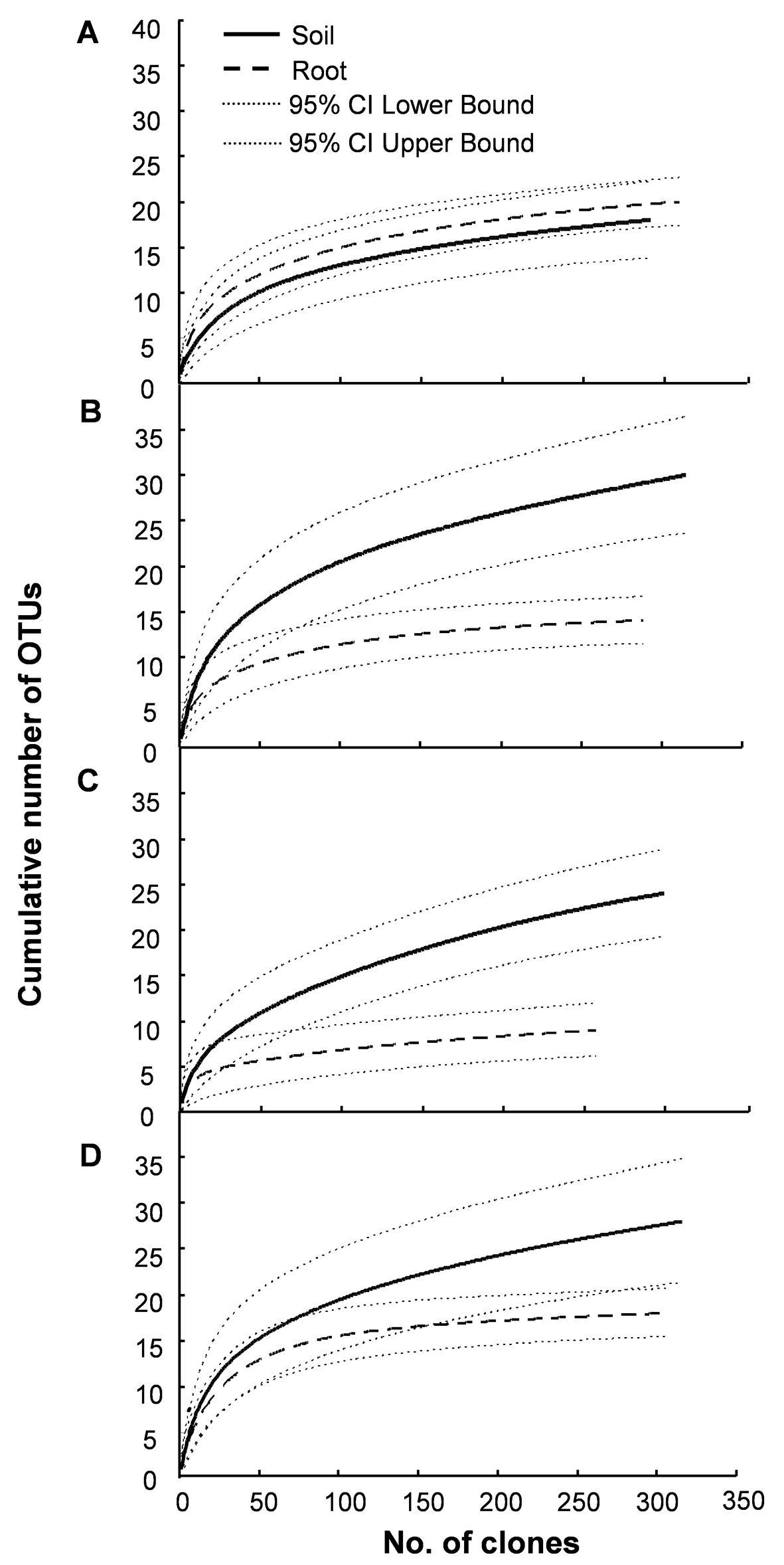

Supplement: Figure S2 — Rarefaction curves for AM fungal OTUs obtained from soil and roots in A: no-warming with no-grazing; B: warming with no-grazing; C: no-warming with grazing; D: warming with grazing. (TIF) [file pone.0076447.s002.tif]
