# Supplementary material for: The Arbuscular Mycorrhizal Fungal Community Response to Warming and Grazing Differs between Soil and Roots on the Qinghai-Tibetan Plateau
Source: PLoS One. 2013 Sep 26;8(9):e76447. doi: 10.1371/journal.pone.0076447 (PMC3784447; doi:10.1371/journal.pone.0076447)
Supplement: Table S1 — Aboveground net primary production (ANPP, g m-2), plant species richness (Richness), soil moisture (SM, %), soil organic carbon (SOC, mg kg-1 DW), soil organic nitrogen (SON, mg kg-1 DW), total nitrogen (TN, % DW), total phosphorus (TP, mg kg-1 DW), and pH value after a 3-year-period of warming and/or grazing. (DOCX) [file pone.0076447.s003.docx]

**Table S1.** Aboveground net primary production (ANPP, g m^-2^), plant species richness (Richness), soil moisture (SM, %), soil organic carbon (SOC, mg kg^-1^ DW), soil organic nitrogen (SON, mg kg^-1^ DW), total nitrogen (TN, % DW), total phosphorus (TP, mg kg^-1^ DW) and pH values after a 3-year-period of warming and/or grazing.

| Variable | C | W | G | WG | Reference |
| --- | --- | --- | --- | --- | --- |
| pH | 7.60±0.14a | 7.61±0.08a | 7.61±0.04a | 7.57±0.15a | Rui *et al*., 2011 |
| SM | 35.25±2.75a | 26.25±2.06b | 35.75±1.50a | 28.00±2.45b | Rui *et al*., 2011 |
| TN | 50.50±3.11a | 50.50±2.08a | 49.75±3.30a | 54.75±3.77a | Rui *et al*., 2011 |
| TP | 777.58±35.25a | 759.75±24.91a | 781.78±16.32a | 758.95±21.81a | Rui *et al*., 2011 |
| SOC | 472.76±106.54a | 453.83±47.32a | 464.67±60.00a | 524.60±111.23a | Rui *et al*., 2011 |
| SON | 26±6.37a | 26.38±5.73a | 24±4.15a | 29.04±11.56a | Rui *et al*., 2011 |
| ANPP | 403.00±11.53b | 569.71±33.88a | 433.80±7.93b | 521.18±37.18c | Wang *et al*., 2012 |
| Richness | 35.25±2.87a | 26.75±1.50b | 27±1.63b | 25±0.82b | Wang *et al*., 2012 |

Values (means ± SD, n = 4) followed by the different letters in the same row indicate significant treatment difference at *P* < 0.05. C, no-warming and no-grazing; G, no-warming and grazing; W, warming and no-grazing; WG, warming and grazing.
