# Supplementary material for: The Arbuscular Mycorrhizal Fungal Community Response to Warming and Grazing Differs between Soil and Roots on the Qinghai-Tibetan Plateau
Source: PLoS One. 2013 Sep 26;8(9):e76447. doi: 10.1371/journal.pone.0076447 (PMC3784447; doi:10.1371/journal.pone.0076447)
Supplement: Table S2 — Abundance (sequence numbers) of arbuscular mycorrhizal (AM) fungal OTUs in soil and roots under no-warming with no-grazing (C), warming with no-grazing (W), no-warming with grazing (G), and warming with grazing (WG). (DOCX) [file pone.0076447.s004.docx]

**Table S2.** Abundance (sequence numbers) of arbuscular mycorrhizal (AM) fungal OTUs in no-warming with no-grazing (C), warming with no-grazing (W), no-warming with grazing (G) and warming with grazing (WG).

| AM fungus | W | | G | | C | | WG | | Family |
| --- | --- | --- | --- | --- | --- | --- | --- | --- | --- |
|  | Soil | Root | Soil | Root | Soil | Root | Soil | Root |  |
| OTU1 | 1 | 0 | 0 | 0 | 0 | 0 | 0 | 0 | Glomeraceae |
| OTU2 | 1 | 0 | 0 | 0 | 0 | 0 | 0 | 0 | Glomeraceae |
| OTU3 | 2 | 0 | 0 | 0 | 0 | 0 | 0 | 0 | Glomeraceae |
| OTU4 | 0 | 0 | 0 | 0 | 1 | 0 | 0 | 0 | Glomeraceae |
| OTU5 | 0 | 0 | 0 | 0 | 2 | 0 | 0 | 0 | Glomeraceae |
| OTU6 | 0 | 0 | 1 | 0 | 0 | 0 | 0 | 0 | Glomeraceae |
| OTU7 | 0 | 0 | 0 | 0 | 0 | 0 | 1 | 0 | Glomeraceae |
| OTU8 | 1 | 0 | 0 | 0 | 0 | 0 | 0 | 0 | Glomeraceae |
| OTU9 | 0 | 0 | 1 | 0 | 0 | 0 | 0 | 0 | Archaeosporaceae |
| OTU10 | 0 | 0 | 0 | 0 | 1 | 0 | 0 | 0 | Diversisporaceae |
| OTU11 | 0 | 0 | 0 | 0 | 1 | 0 | 0 | 0 | Diversisporaceae |
| OTU12 | 0 | 0 | 0 | 0 | 0 | 0 | 2 | 0 | Claroideoglomeraceae |
| OTU13 | 0 | 0 | 0 | 0 | 0 | 0 | 1 | 0 | Glomeraceae |
| OTU14 | 0 | 0 | 0 | 0 | 0 | 0 | 1 | 0 | Glomeraceae |
| OTU15 | 0 | 0 | 0 | 0 | 0 | 0 | 1 | 0 | Glomeraceae |
| OTU16 | 0 | 0 | 0 | 0 | 0 | 0 | 1 | 0 | Claroideoglomeraceae |
| OTU17 | 0 | 1 | 0 | 0 | 0 | 0 | 0 | 0 | Glomeraceae |
| OTU18 | 0 | 0 | 0 | 0 | 0 | 1 | 0 | 0 | Gigasporaceae |
| OTU19 | 0 | 0 | 0 | 0 | 0 | 0 | 0 | 1 | Gigasporaceae |
| OTU20 | 0 | 0 | 0 | 1 | 0 | 0 | 0 | 0 | Gigasporaceae |
| OTU21 | 0 | 0 | 0 | 0 | 0 | 0 | 0 | 2 | Gigasporaceae |
| OTU22 | 0 | 0 | 0 | 0 | 0 | 2 | 0 | 0 | Glomeraceae |
| OTU23 | 0 | 0 | 0 | 0 | 0 | 6 | 0 | 0 | Glomeraceae |
| OTU24 | 2 | 0 | 1 | 0 | 0 | 0 | 11 | 19 | Glomeraceae |
| OTU25 | 30 | 13 | 44 | 34 | 172 | 60 | 71 | 118 | Gigasporaceae |
| OTU26 | 0 | 14 | 0 | 42 | 0 | 2 | 26 | 47 | Glomeraceae |
| OTU27 | 12 | 30 | 1 | 145 | 10 | 21 | 1 | 13 | Glomeraceae |
| OTU28 | 2 | 0 | 1 | 0 | 0 | 0 | 31 | 0 | Glomeraceae |
| OTU29 | 31 | 28 | 2 | 3 | 0 | 21 | 0 | 22 | Glomeraceae |
| OTU30 | 32 | 6 | 128 | 0 | 27 | 35 | 36 | 14 | Diversisporaceae |
| OTU31 | 6 | 3 | 0 | 0 | 18 | 6 | 5 | 10 | Glomeraceae |
| OTU32 | 26 | 0 | 17 | 0 | 7 | 0 | 11 | 14 | Glomeraceae |
| OTU33 | 21 | 0 | 0 | 0 | 0 | 0 | 0 | 0 | Glomeraceae |
| OTU34 | 6 | 0 | 0 | 26 | 0 | 5 | 1 | 0 | Glomeraceae |
| OTU35 | 47 | 0 | 44 | 0 | 0 | 0 | 26 | 0 | Glomeraceae |
| OTU36 | 0 | 0 | 0 | 0 | 0 | 0 | 5 | 0 | Glomeraceae |
| OTU37 | 50 | 121 | 9 | 4 | 7 | 0 | 25 | 0 | Glomeraceae |
| OTU38 | 11 | 3 | 0 | 0 | 0 | 6 | 4 | 8 | Glomeraceae |
| OTU39 | 0 | 0 | 3 | 0 | 0 | 0 | 0 | 4 | Glomeraceae |
| OTU40 | 1 | 0 | 0 | 0 | 0 | 0 | 0 | 0 | Glomeraceae |
| OTU41 | 7 | 0 | 0 | 0 | 0 | 0 | 3 | 0 | Glomeraceae |
| OTU42 | 4 | 0 | 0 | 0 | 0 | 75 | 0 | 0 | Glomeraceae |
| OTU43 | 6 | 5 | 1 | 0 | 3 | 0 | 9 | 7 | Glomeraceae |
| OTU44 | 0 | 0 | 1 | 0 | 1 | 0 | 18 | 0 | Glomeraceae |
| OTU45 | 0 | 0 | 0 | 0 | 0 | 0 | 0 | 5 | Glomeraceae |
| OTU46 | 7 | 0 | 0 | 0 | 2 | 2 | 3 | 7 | Glomeraceae |
| OTU47 | 0 | 0 | 0 | 0 | 4 | 0 | 0 | 0 | Glomeraceae |
| OTU48 | 1 | 0 | 16 | 0 | 0 | 0 | 0 | 0 | Glomeraceae |
| OTU49 | 3 | 0 | 2 | 0 | 0 | 0 | 0 | 0 | Diversisporaceae |
| OTU50 | 1 | 0 | 0 | 0 | 0 | 0 | 16 | 0 | Glomeraceae |
| OTU51 | 0 | 0 | 0 | 0 | 16 | 1 | 0 | 0 | Glomeraceae |
| OTU52 | 1 | 0 | 1 | 0 | 0 | 43 | 2 | 0 | Claroideoglomeraceae |
| OTU53 | 0 | 1 | 1 | 1 | 0 | 2 | 1 | 0 | Glomeraceae |
| OTU54 | 0 | 0 | 0 | 0 | 0 | 6 | 0 | 10 | Glomeraceae |
| OTU55 | 1 | 0 | 3 | 0 | 0 | 13 | 0 | 0 | Glomeraceae |
| OTU56 | 1 | 0 | 2 | 0 | 15 | 0 | 0 | 5 | Glomeraceae |
| OTU57 | 0 | 0 | 13 | 1 | 0 | 1 | 0 | 0 | Glomeraceae |
| OTU58 | 0 | 0 | 3 | 0 | 0 | 0 | 0 | 0 | Glomeraceae |
| OTU59 | 0 | 2 | 0 | 0 | 0 | 0 | 0 | 0 | Glomeraceae |
| OTU60 | 0 | 4 | 0 | 0 | 0 | 0 | 0 | 0 | Glomeraceae |
| OTU61 | 1 | 0 | 0 | 0 | 3 | 1 | 0 | 0 | Gigasporaceae |
| OTU62 | 0 | 22 | 0 | 0 | 1 | 0 | 1 | 1 | Gigasporaceae |
| OTU63 | 1 | 0 | 0 | 0 | 0 | 0 | 3 | 0 | Glomeraceae |
| OTU64 | 0 | 0 | 2 | 0 | 0 | 0 | 0 | 0 | Glomeraceae |
| OTU65 | 0 | 0 | 2 | 0 | 0 | 0 | 0 | 0 | Diversisporaceae |
| Sum of sequences | 315 | 253 | 299 | 257 | 291 | 309 | 316 | 307 |  |
| Total OTU number | 30 | 14 | 24 | 9 | 18 | 20 | 28 | 18 |  |
