# Supplementary material for: The Arbuscular Mycorrhizal Fungal Community Response to Warming and Grazing Differs between Soil and Roots on the Qinghai-Tibetan Plateau
Source: PLoS One. 2013 Sep 26;8(9):e76447. doi: 10.1371/journal.pone.0076447 (PMC3784447; doi:10.1371/journal.pone.0076447)
Supplement: Table S3 — Arbuscular mycorrhizal fungal richness of Glomeraceae, Claroideoglomeraceae, Diversisporaceae and Gigasporaceae in soil and roots under no-warming with no-grazing (C), warming with no-grazing (W), no-warming with grazing (G), and warming with grazing (WG). (DOCX) [file pone.0076447.s005.docx]

**Table S3.** Arbuscular mycorrhizal fungal OTU richness of Glomeraceae, Claroideoglomeraceae, Diversisporaceae and Gigasporaceae in soil and root under no-warming with no-grazing (C), warming with no-grazing (W), no-warming with grazing (G) and warming with grazing (WG).

| Family | Soil | | | |  | Root | | | |
| --- | --- | --- | --- | --- | --- | --- | --- | --- | --- |
|  | C | W | G | WG |  | C | W | G | WG |
| Glomeraceae | 4.25±1.71a | 9.75±4.27a | 6.25±2.22a | 7.50±2.52a |  | 5.75±1.50a | 5.00±1.83a | 2.75±1.50a | 5.75±2.50a |
| Claroideoglomeraceae | 0.00±0.00a | 0.25±0.50a | 0.25±0.50a | 0.75±1.50a |  | 0.50±0.58a | 0.00±0.00a | 0.00±0.00a | 0.00±0.00a |
| Diversisporaceae | 1.00±1.41a | 1.25±0.50a | 1.50±0.58a | 0.25±0.50a |  | 1.25±0.50a | 0.50±1.00a | 0.75±0.50a | 1.75±0.50a |
| Gigasporaceae | 2.00±0.82a | 1.00±0.82a | 0.75±0.50a | 1.25±0.50a |  | 1.50±0.58a | 0.50±1.00a | 0.75±0.50a | 1.75±0.50a |

Data (means ± SD, n = 4) followed by different letters in the same row indicate significant differences among treatments at *P* < 0.05.
